# Differences in Velocities of Crucial Body Segments while Executing Roundhouse Kicks for Both Sides

**DOI:** 10.5114/jhk/159451

**Published:** 2023-01-20

**Authors:** Jacek Wąsik, Dariusz Mosler, Dorota Ortenburger, Tomasz Góra, Robert Podstawski

**Affiliations:** 1 Department of Kinesiology and Health Prevention, Jan Dlugosz University of Czestochowa, Czestochowa, Poland.; 2 Department of Physiotherapy, School of Public Health, University of Warmia and Mazury in Olsztyn, Olsztyn, Poland

**Keywords:** kicking velocity, dynamic balancing, taekwon-do, movement analysis, kicking kinematics

## Abstract

Lower limb kinematics of the roundhouse kick is a well-known topic studied by many researchers. However, there is a lack of data about the velocity of the core and upper limbs during the execution of this technique. The aim of this study was to evaluate the differences in velocities of all crucial body segments while executing roundhouse kicks for both sides of the body. Thirteen elite taekwon-do athletes participated in this study. They performed kicks to a table tennis ball three times using each leg. The spatial-temporal data of markers placed on toes, knees, hips, shoulders, elbows, hands, and sternum were captured with the use of the Human Motion Lab equipment composed of 10 infrared cameras NIR Vicon MX-T40. There were statistical differences in the maximal velocity of the sternum and opposite shoulder. There were different correlations between the time of acquiring maximal velocities of specific body segments and the maximal velocity of the toe marker for each kicking side. Higher correlations were observed for the left kick despite the participant’s declaration of their preference for the right leg. The obtained results facilitate the conclusion that small non-resistant targets require different motor control depending on the kicking side, despite not revealing significant differences between maximal velocity. While such an indicator could be perceived as a suitable benchmark of an athlete’s performance, more detailed analysis seems to be required for a better understanding of martial arts techniques.

## Introduction

A roundhouse kick (in taekwon-do terminology: dollyo chagi) is one of the most frequently described martial arts techniques in biomechanics-related literature (Estevan et al., 2012, 2015; Wąsik et al., 2021). The literature indicates that the roundhouse kick is the most popular technique used in official taekwon-do combat competitions (Matsushigue et al., 2009; Pyciarz, 2011).

A points-based system in martial arts competitions, such as karate or taekwon-do, does not allow the intentional knock-out of an opponent. A regular technique which hits an opponent gives points to the competitor. Finally, the overall score determines the winner (Hoelbling et al., 2020). Such competition criteria require fast and precise strikes, which allows the competitor to acquire points faster than the opponent. Therefore, athletes need to shorten the time between the first sign of beginning a strike to the contact with the target (Hölbling et al., 2017). This task is very demanding, especially for more complex techniques or combinations of techniques.

The relation between the shortest kick trajectory and the maximal velocity of a roundhouse kick is not known. Many researchers have measured the maximal velocity of a roundhouse kick for different martial arts athletes, thus establishing the maximal velocity at 13.24 m/s for muay thai, 13.66 m/s for karate (Gavagan and Sayers, 2017), 17.35 m/s for kickboxing (Aandahl et al., 2018), and close to 16 m/s for taekwon-do (Moreira et al., 2016).

The time of technique execution and the repeated maximal velocity results could be a promising benchmark indicating the athlete’s performance and the level of preparedness (Pozo et al., 2011). Striking techniques are performed in an open kinematic chain in a throw-like pattern of movement which was determined by Kim et al. (2011), while also describing the intra-limb coordination of roundhouse kicks. Falco et al. (2013) determined that muscle activation and the peak velocities of specific segments followed proximal to distal patterns. In sports science, in the past decade there has been an increased interest in the control of the central parts of the body (Barbado et al., 2016; Reed et al., 2012; Wąsik, 2012; Zouita Ben Moussa et al., 2020). The importance of core stability is said to guarantee proper distal mobility (Okada et al., 2011). Even if the phenomenon of balance and stability is the subject of research, it is perceived as a separate aspect of kinesiology. Therefore, in the biomechanics research of roundhouse kick kinematics, there is a gap in a full body motion analysis of separate core and upper limb segments alongside the lower extremity. Such analysis is constantly performed in motion capture for special effects in games and movies, but the obtained data are not analysed for sporting performance purposes. Nevertheless, the relation between separate body segments could provide further understanding of how to achieve higher kick velocity by improving technique.

The roundhouse kick is a sport technique with a rotational motion of the body. According to the second law of Newton’s dynamics for rotational motion, we know that the torque is responsible for the angular momentum (Crothers, 1992). This means that the wider the swing of the arms during trunk rotation, the greater the power will be generated and the angular momentum will be greater accordingly. In that manner, the power generated by the lower limbs during the execution of the kick will be higher. This premise facilitates the assumption that to fully understand roundhouse kick kinematics, a full body analysis of an athlete is required.

The aim of this study was to determine how the velocity of chosen body segments of taekwon-do athletes affected the maximal velocity of the foot during the execution of a roundhouse kick and to determine the similarities and differences between both kicking lower extremities.

## Methods

### Participants

There were 13 participants in this study, all of whom were elite taekwon-do ITF (International Taekwon-do Federation) athletes (aged: 22.6 ± 6.28 years; body mass: 73.76 ± 10.16 kg; body height: 177.69 ± 6.78 cm), with at least 4 years of fighting experience. Participants declared that their preferred kicking leg was the right one.

### Measures

For the purpose of this study, a stereo photogrammetric method of motion capture was used. The study was conducted in a Human Motion Lab (HML) equipped with 10 infrared cameras NIR Vicon MX-T40 with a resolution of 4 megapixels and a 10-bit grey scale. This system is capable of capturing 370 frames per second. In order to analyse the motion of all key segments of the body, ten markers were used. Two markers were placed on the side of the proximal phalanx great toe (rtoe, ltoe), on the side of the knee (rkne, lkne – r stands for right side, l for the left side), the anterior superior iliac spine (rasi, lasi), the sternum (strn), the acromial end of clavicle (rclo, lclo), on the side of the elbows (relb, lelb), and both hands (rhno, lhno). Spatial-temporal data of marker displacement in time function for X,Y, Z plane were captured and stored in c3d format data. With the use of algorithms written in python with the use of pyomeca library (Martinez et al., 2020), the resultant velocity was computed for all markers and the maximal velocity for each motion was determined.

### Design and Procedures

**Figure 1:**
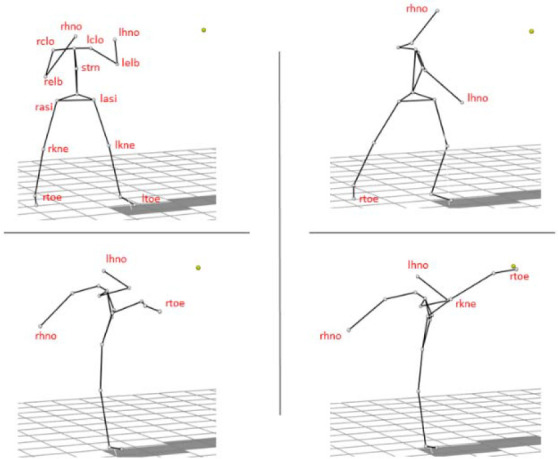
Visualization of technique execution and registered markers placement. Two markers were placed on the side of the proximal phalanx great toe (rtoe, ltoe), on the side of the knee (rkne, lkne – r stands for the right side, l for the left side), anterior superior iliac spine (rasi, lasi), sternum (strn), acromial end of the clavicle (rclo, lclo), side of the elbows (relb, lelb), and both hands (rhno, lhno).

Participants executed a taekwon-do version of the roundhouse kick. It is a circular motion kick performed from a side stance. Upon raising the kicking leg, there is rotation on the supporting leg, bending in the knee and the hip joint in the kicking leg, together with torso rotation toward a target with the corresponding side of the body and finally knee extension to reach the target with the foot. All strikes were performed on a table tennis ball hanging on a string from the ceiling. The target was non-resistant and athletes performed 3 strikes for each side of the body. Overall, 78 kicks were captured. The marker placement and visualization of the movement are presented in Figure 1.

Participants were told to measure their subjective distance from the target by marking the kicking move before the actual attempt to adjust it to their height. Fixing one standing line for measurements resulted in unbiased data due to the height differences. Participants were told to kick as fast as they could and hit the target. If the target was missed, the trial was continued until three hits were captured. There was no starting signal and participants were informed that the motion capture had started and they were free to start when they felt ready.

### Statistical Analysis

The mean and standard deviation were computed for all the obtained maximal velocities of the markers. The correlations between the variables (markers and anthropometric data) were determined by the Pearson correlation coefficient. Likewise, the differences between both kicking sides were determined with the use of the univariate analysis of (ANOVA). The level of significance was set at *p* < 0.05. All statistical computations were performed with the use of Statistica 12 (Statsoft, Hamburg, Germany).

### Ethics

The Human Subjects Research Committee of the host University scrutinized and approved the test protocol as fulfilling the criteria of Ethical Conduct for Research Involving Humans (Bioethics Committee at the University of Rzeszów, No. 2/6/2017). All subjects in the study were informed of the testing procedures and participated in the data collection voluntarily.

## Results

**Figure 2:**
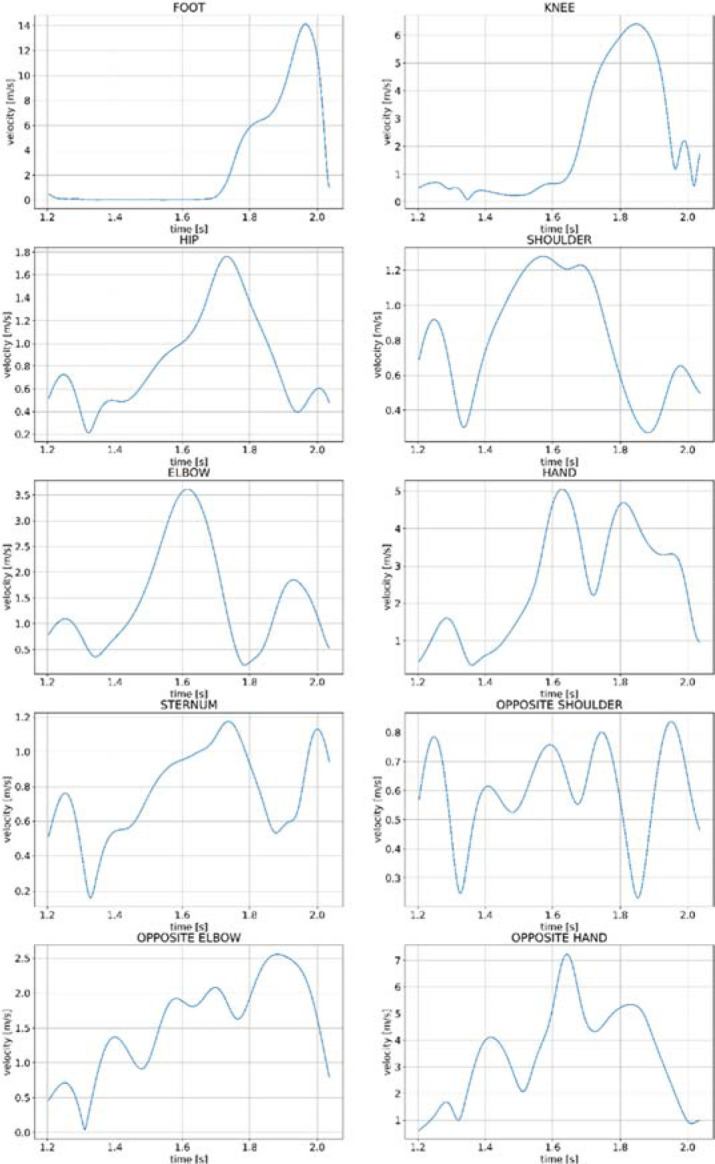
Sample visualisation of selected markers velocity during roundhouse kick execution.

The highest maximal velocity was obtained for toe markers with a higher mean velocity for the left leg (12.94 m/s and 13.19 m/s, respectively). Secondly, there were knee markers with a mean of 6.20 m/s, a hand marker of the opposite side to the kicking leg (5.75 m/s) and a hand on the kicking side of the body (5.40 m/s). The minimal obtained velocity of the toe marker was 8.85 m/s and the maximum value was 16.58 m/s (Table 1). Figure 2 presents a sample graph of changes in velocities of a specific marker in time. The closest time of maximal velocity occurrence to the toe marker is the opposite shoulder (in that case left). The corresponding part of the body represents a proximal to distant pattern of maximal velocity occurrence, while the opposite part of the body presents a distal to proximal pattern and the opposite shoulder achieved maximal velocities after the opposite elbow and the opposite hand (Figure 2).

**Table 1:** Maximal velocities of chosen makers placed on the athletes’ bodies (m/s).

Marker velocity (m/s)	Right		Left		Total			
	Mean	SD	Mean	SD	Mean	SD	Min	Max
toe	12.94	2.40	13.193	2.271	13.07	2.16	8.85	16.58
knee	6.26	0.93	6.139	0.859	6.20	0.87	4.94	8.08
hip	1.96	0.28	1.877	0.375	1.92	0.32	1.45	2.48
clavicle	1.54	0.18	1.490	0.302	1.52	0.19	1.26	1.92
elbow	3.32	0.78	3.315	0.895	3.32	0.80	2.32	5.19
hand	5.38	1.50	5.408	1.642	5.40	1.46	2.48	7.62
sternum	1.55	0.25	1.397	0.202	1.47	0.19	1.22	1.77
opposite clavicle	1.46	0.22	1.253	0.223	1.36	0.19	1.10	1.81
opposite elbow	3.24	0.78	2.962	0.760	3.10	0.68	2.03	4.07
opposite hand	6.16	2.44	5.341	2.851	5.75	2.35	2.96	11.38

SD – standard deviation, Min – minimum, Max – maximum

Despite the differences in mean values of maximal velocities of distant markers, ANOVA did not reveal any significant differences between the left and right roundhouse kicks (the side was set as a categorical variable). However, statistically significant differences were revealed for the categorical variable sternum and the opposite to the kicking side clavicle at a significance level of *p* < 0.01 (Table 2).

**Table 2:** ANOVA results of velocities of different parts by the kicking side variable.

Marker velocity	SS	MS	F	*p*
toe	1.242	1.242	0.212	0.647
knee	0.265	0.265	0.302	0.584
hip	0.124	0.124	0.937	0.336
clavicle	0.055	0.055	0.729	0.396
elbow	0.001	0.001	0.002	0.968
hand	0.014	0.014	0.005	0.944
sternum	0.469	0.469	7.394	0.008*
opposite clavicle	0.864	0.864	12.762	0.001*
opposite elbow	1.485	1.485	2.132	0.148
opposite hand	13.076	13.076	1.733	0.192

SS – sum of squares; MS – mean square; F – Fisher statistic value; p – significance level value; * - statistically significant value

Almost all pairs of markers showed significant correlations between each other. The highest correlation was revealed for the hand-toe and the hand-knee pairing on the kicking side of the body (r = 0.782). The second strongest relation was between the hand and the opposite elbow (r = 0.770) and the knee opposite the elbow (0.757). Subsequently, there were high correlation values of the hips in a pairing with the sternum (r = 0.747) and the clavicle of the corresponding side (r = 0.732). Finally, there was a high correlation between the toe and the knee marker of the kicking leg (r = 0.719). The remaining values express only moderate correlations. Only three pairs failed to reveal statistically significant relations. The first one was the toe-clavicle pair, while the second one included the clavicle with the opposite elbow and with opposite hands. A detailed correlation matrix is presented in Table 3.

**Table 3:** Spearman’s correlation matrix between maximal velocities of each marker pair.

Marker velocity	toe	knee	hip	clavicle	elbow	hand	sternum	opposite clavicle	opposite elbow
knee	0.719								
hip	0.344	0.622							
clavicle	0.090	0.322	0.732						
elbow	0.491	0.702	0.624	0.534					
hand	0.782	0.782	0.520	0.228	0.660				
sternum	0.269	0.495	0.747	0.664	0.544	0.485			
opposite clavicle	0.452	0.603	0.518	0.288	0.462	0.528	0.614		
opposite elbow	0.693	0.757	0.392	0.094	0.395	0.770	0.446	0.625	
opposite hand	0.580	0.678	0.446	0.068	0.548	0.673	0.426	0.617	0.724

There were no straightforward tendencies in the correlation between the maximal velocities of the roundhouse kicks and anthropometric data. Only for the preferred leg (right), there was a negative moderate correlation between maximal velocity of the toe marker and age (r = -0.582). For the left kicks, a moderate significant correlation was revealed between the clavicle marker and body height (r = 0.573) as well as body mass (r = 0.644). In summary, considering mean value of both sides of the body, only body mass was significantly correlated with the clavicle (r = 0.694). Beside those mentioned pairs, all other correlations were low and statistically non-significant (Table 4).

**Table 4:** Correlation matrix between mean maximal velocities values of each marker and anthropometric data.

Marker velocity	right			left			total		
	age	height	weight	age	height	weight	age	height	weight
toe	−0.582	−0.096	−0.298	−0.188	−0.182	−0.178	−0.421	−0.149	−0.258
knee	−0.046	−0.067	0.005	0.045	−0.031	−0.043	−0.003	−0.051	−0.018
hip	0.264	0.192	0.369	0.366	0.390	0.521	0.331	0.314	0.469
clavicle	0.403	−0.128	0.428	0.241	0.573	0.644	0.370	0.387	0.694
elbow	0.032	−0.335	0.076	0.192	−0.161	0.230	0.124	−0.255	0.167
hand	−0.251	0.084	0.061	0.006	−0.346	0.047	−0.125	−0.151	0.058
sternum	0.024	0.096	0.091	0.293	0.334	0.509	0.170	0.237	0.326
opp. clavicle	−0.131	−0.111	−0.141	0.159	−0.034	−0.016	0.017	−0.084	−0.092
opp. elbow	−0.219	0.160	0.106	0.030	−0.281	−0.168	−0.108	−0.065	−0.033
opp. hand	−0.117	0.019	0.034	−0.006	−0.471	−0.340	−0.065	−0.276	−0.189

## Discussion

The obtained results of maximal velocity of markers placed on the leg are in line with the results of similar research conducted by other authors. The mean velocity of the toe marker was 13.07 ± 2.16 m/s with the highest achieved velocity of 16.58 m/s. Although participants in other studies performed roundhouse kicks without a physical target or training equipment, such as pads or shields, they reached velocities of 12.89 m/s in the study conducted by Estevan et al. (2013), while in Gavagan et al.’s (2017) study, it was 14.66 m/s and in Kim et al.’s (2011) study, it was 14.7 m/s, whereas in the study by Aandahl et al. (2018), athletes reached values up to 17.35 m/s. None of those researchers, however, considered upper limb or torso marker velocities.

The lack of significant differences between both kicking sides for the distal markers placed on the limbs indicates the uniformity of the performance level for both legs of athletes. This phenomenon could be explained by the training type of martial arts practitioners. In the traditional type of taekwon-do training, the techniques are practised for both sides of the body with an even load (Pop et al., 2013). Athletes have lateral preferences during competitions (Loffing et al., 2014; Mala et al., 2019), but under laboratory conditions of motion capture, this was not evidenced, which is also in line with previous research (Wąsik et al., 2016). The bilateral practice of techniques is an important part of training, which leads to the execution of a complex combination of movements. Psychologists indicate that the rhythm and repeatability of movements is beneficial for focusing, which gives better results for mental training and satisfaction levels (Bu et al., 2010; MacPherson et al., 2009). There are premises to indicate that the increased coordination levels and abilities of non-dominant limbs facilitate adaptive neural changes (Stöckel and Weigelt, 2012).

The obtained results confirm significant relationships of the velocity of the toe and knee markers with the execution of the roundhouse kick (r = 0.719). From the sample analysis, the movement sequence generally starts with the hands, then the hips and the knee flows and the maximal velocity of the foot is reached at the end, just before contact with the target. A strong correlation between the hands and lower limb segements (r = 0.782) indicates a strong influence of the upper limb movement on the velocity of lower limbs. Moreover, the high correlation between the hip and the clavicle marker (shoulder girdle) (r = 0.732) or sternum (r = 0.747) on the kicking side indicates girdle additive value of the torso rotation in the execution of the movement. It could be concluded that the initial upper limb swing followed by trunk rotation are key elements in the execution of the roundhouse kick. There is a lack of supporting evidence in the field of the biomechanics of roundhouse kicks on upper limbs and the importance of core muscles. Nevetherless, there are substantial amounts of data from the field of sport science studies indicating a better performance of the distal parts supported by strong core muscles (Hibbs et al., 2008). The similarity in a throw-like pattern of the roundhouse kick could be compared to throwing a baseball or a javelin, which requires sufficient core strength to generate power for quick movements (Kim et al., 2014; Lust et al., 2009).

ANOVA revealed that there were differences in maximal velocities of the opposite clavicle and the sternum, despite the lack of the afore-mentioned distal differences. Perhaps there are different movement patterns for both sides of the body, which are compensated into the other segments, which leads to a uniform performance for both sides. The key explanation may be in the type of the target. A small, non-resistant target is demanding in terms of precision and coordination, which is different from the usual practice targets. There are proven differences in maximal velocities of different martial arts techniques depending on the type of the target (Wąsik et al., 2016; Wąsik and Góra, 2016).

Non-conclusive analysis of the correlation of anthropomorphic variables with the kinematic variables indicates that age, height and weight are not the most significant factors for the execution of martial arts techniques in terms of maximum velocity. Perhaps further research with different targets of the central part of the body, velocity and force analysis will contribute to the knowledge about this phenomena (Zemková, 2018, 2019).

This study extends the knowledge of biomechanic dependencies of martial arts performance. Our findings present another stage of kinematic dependencies research and could serve as a framework of reference for other researchers, thus encouraging them to perform full body analysis, instead of focusing solely on the lower limb kinematics.

## Conclusions

The present study indicates that during the execution of the roundhouse kick by taekwondo ITF athletes, there is no lateralization effect of the kicking sides besides the central body parts. The data with no lateralization effect besides the central body parts of the tested athletes could be considered from the perspective of the concept, which states that complex motor tasks could be changed at the sensory-motor level during its execution, depending on the needs and circumstances. There is a strong relation between maximal velocity of the foot and both hands, foot and shoulder girdle, as well as between the hips and knees of the kicking side of the body. More emphasis should be put on a full body analysis, as well as the kicking force analysis for a better understanding of the lateralization phenomenon, together with a full kinematic chain of the techniques executed.
